# Antibiotic exposure in prenatal and early life and risk of juvenile idiopathic arthritis: a nationwide register-based cohort study

**DOI:** 10.1136/rmdopen-2023-003333

**Published:** 2023-08-30

**Authors:** Sigrid Hestetun, Svend Andersen, Helga Sanner, Ketil Størdal

**Affiliations:** 1 Department of Rheumatology, Oslo University Hospital, Oslo, Norway; 2 Institute of Clinical Medicine, Faculty of Medicine, University of Oslo, Oslo, Norway; 3 Department of Paediatrics, Vestfold Hospital Trust, Tønsberg, Norway; 4 Department of Health Sciences, Oslo New University College, Oslo, Norway; 5 Division of Paediatric and Adolescent Medicine, Oslo University Hospital, Oslo, Norway; 6 Paediatric Research Institute, Faculty of Medicine, University of Oslo, Oslo, Norway

**Keywords:** arthritis, juvenile, autoimmune diseases, epidemiology

## Abstract

**Objectives:**

Early antibiotic exposure influences the gut microbiota which is believed to be involved in the pathogenesis of juvenile idiopathic arthritis (JIA). We aimed to investigate the association between systemic antibiotics in prenatal and early life and risk of JIA.

**Methods:**

We conducted a register-based cohort study including all children born in Norway from 2004 through 2012. The children were followed until 31 December 2020. Main exposures were dispensed antibiotics to the mother during pregnancy and to the child during 0–24 months of age. The outcome was defined by diagnostic codes indicating JIA. Multivariate logistic regression analyses were performed to estimate the association between antibiotic exposure and JIA.

**Results:**

We included 535 294 children and their mothers in the analyses; 1011 cases were identified. We found an association between exposure to systemic antibiotics during 0–24 months and JIA (adjusted OR (aOR) 1.40, 95% CI 1.24 to 1.59), with a stronger association for >1 course (aOR 1.50, 95% CI 1.29 to 1.74) vs 1 course (aOR 1.31, 95% CI 1.13 to 1.53). Subanalyses showed significant associations in all age periods except 0–6 months, and stronger association with sulfonamides/trimethoprim and broad-spectrum antibiotics. There was no association between prenatal antibiotic exposure and JIA.

**Conclusions:**

The novel observation of no association with prenatal antibiotic exposure and JIA suggests that the association between antibiotics in early life and JIA is unlikely to be confounded by shared family factors. This may indicate that exposure to antibiotics in early life is an independent risk factor for JIA.

**WHAT IS ALREADY KNOWN ON THIS TOPIC**

- Antibiotic exposure in early life can have lasting impact on the composition and diversity of the gut microbiota.
- While environmental risk factors for juvenile idiopathic arthritis (JIA) are largely unknown, early life antibiotics and gut microbiota have been linked to disease development.

**WHAT THIS STUDY ADDS**

- In our nationwide study, antibiotic exposure during age 0–24 months but not prenatal antibiotics was associated with later JIA development.
- The novel finding of no association with prenatal antibiotic exposure suggests that the association between antibiotic exposure in childhood and JIA is unlikely to be confounded by shared family factors.

**HOW THIS STUDY MIGHT AFFECT RESEARCH, PRACTICE OR POLICY**

- Our findings suggest that antibiotic exposure in early life is an independent risk factor for JIA.
- Future studies should aim to capture different infectious agents and antibiotics to separate these potential risk factors.
- Biomarkers of the gut microbiome before development of JIA would strengthen a potential causal link.

## Introduction

Juvenile idiopathic arthritis (JIA) is the most common chronic inflammatory rheumatic condition of childhood and is usually categorised into seven clinically heterogeneous subtypes with chronic arthritis as a common characteristic.^1^ A pooled annual incidence rate of 7.8/100 000 among Caucasians has been reported with the highest incidence in Europe, especially Nordic countries (15–22.6/100 000).^2–4^ The aetiology of JIA is multifactorial with both genes and environmental factors contributing.^5 6^ However, since there is a lack of high-quality studies concerning environmental risk factors, knowledge is limited.^5–7^

There is an increasing interest on the early life gut microbiota and its role in the development of autoimmune diseases, including JIA.^7–9^ The gut microbiota develops rapidly during the first years of life and stabilises at around three years of age.^10^ The main determinants of the gut microbiota are mode of delivery, infant feeding and exposure to antibiotics.^11^ Exposure to systemic antibiotics before a stable mature microbiome is established may have a long-lasting influence on the composition and diversity of the gut microbiota of the child.^12^

There is an interplay between the gut microbiota and the development of the immune system. Influences on the immune system induced by the microbiota early in life can be lasting, with consequences for disease development later in life.^7^ Children with JIA were found to have an altered composition and a lower diversity in their gut microbiota compared with healthy children.^13^ Cross-sectional studies are, however, insufficient to study causality, and it remains unclear whether this is involved in the aetiology of the disease.^13^ Interestingly, a recent cohort study demonstrated an altered gut microbiota as early as in infancy in 12 children who later developed JIA.^14^

Some studies have found a positive association between exposure to antibiotics in early life and JIA.^15–18^ However, the studies have limitations including small sample sizes, case–control designs with high risk of recall bias, significant loss to follow-up, lack of exposure data from the neonatal period, and relatively low age at JIA diagnoses, factors that may limit the generalisability of the studies. The association of prenatal antibiotic exposure on the risk of developing JIA is mainly unexplored.^17^ Thus, the objective of our study was to investigate the association between exposure to systemic antibiotics in prenatal and early life, with the risk of developing JIA in a cohort study.

## Methods

### Study population and design

This study was a nationwide register-based cohort study from Norway. All children born between 1 January 2004 and 31 December 2012 and registered in the Medical Birth Register of Norway (MBRN) were included. MBRN is a national health registry containing information about all births in Norway, and the register is based on mandatory reporting.^19^ We linked data from MBRN to relevant population-based registers on an individual level using the unique national identification (ID) number.

### Outcome

From 2008, The Norwegian Patient Registry (NPR) has received data with personal ID numbers from all Norwegian public hospitals and specialists with public funding,^20^ and thus captures virtually all patients with JIA. JIA cases were defined by a combination of International Classification of Diseases (ICD) codes (ICD-10 codes M08 Juvenile arthritis and M09 Juvenile arthritis in diseases classified elsewhere like juvenile arthritis in psoriasis and inflammatory bowel disease) reported to NPR between 1 January 2008 and 31 December 2020.

Children with either ≥2 M08, ≥2 M09 or 1 M08 combined with 1 M09 ICD-10 codes were classified as cases. In addition, children with only 1 M08 or 1 M09 were classified as cases if the year of diagnosis was 2020 because this was the last year with data from NPR and it is likely that some of these children only had only one visit before the end of the year.

We had no data on exact dates of registrations, but cumulative data for registrations each year from 2008 and onwards. Year of onset was defined as the first M08 or M09. In cases where M08/M09 were preceded by a code of M13 (other arthritis), the first entry of M13 was used as onset. Due to missing ID numbers on data from NPR before 2008,^20^ exact year of diagnosis was not available for children diagnosed 2004–2007. Since we only had birth year and birth month available, a replacement birthday on the 15th in each month was constructed to calculate age at exposure and outcome.

### Exposure

For data on exposure to antibiotics, the children and their mothers were linked to The Norwegian Prescription Database (NorPD). Prenatal exposure was defined as any dispensed systemic antibiotic to the mother from a pharmacy during pregnancy. Further, child antibiotic exposure was defined as any dispensed systemic antibiotic to the child from a pharmacy during 0–24 months of age. In addition, data on systemic parenteral antibiotics given during the neonatal period in hospital were available from MBRN and included in the main exposure.

In Norway, antibiotics are only available by prescription. All prescribed drugs dispensed from Norwegian pharmacies are registered at the individual level in NorPD and classified after Anatomic Therapeutic Classification codes.^21^ The different types of antibiotics were categorised into five groups. In approximately 30% of the prescriptions, there was a lack of ID numbers, precluding the individual linkage.

### Other variables

From MBRN we included child’s sex, birth weight, season of birth, year of birth, mode of delivery, pre-eclampsia, maternal age, parity and smoking in pregnancy. From Statistics Norway (SSB), maternal educational level by end of follow-up was included.

### Patient and public involvement

Patient and public involvement was not considered relevant in this registry-based study.

### Statistical analysis

We performed χ^2^ tests to investigate associations between categorical variables. Further, we performed multivariate logistic regression analyses to estimate the OR for JIA by antibiotic exposure prenatally or during 0–24 months of age. The analyses were performed with cluster correction to adjust for correlation between siblings. In the main analyses, we adjusted for sex (adjusted OR (aOR) because JIA is more common in girls than in boys, and boys more often receive antibiotics in early life.^7 21^ Further, we performed subanalyses on number of dispensed antibiotics, exposure in different time periods and on types of antibiotics dispensed. In secondary models the effects of adjusting for the following potential confounders were assessed: prenatal antibiotic exposure, season of birth, year of birth, mode of delivery, birth weight, pre-eclampsia, maternal age, maternal parity, maternal educational level and maternal smoking during pregnancy.

Since the most common subtypes of JIA have a biphasic peak in age at onset with the first peak already between 2 and 4 years,^7^ we could not exclude an overlap between the timing of antibiotic exposure and disease onset in some children. We, therefore, performed a sensitivity analysis excluding children with JIA onset before 3 years of age, and all children with JIA born 2004–2006 since the exact year of diagnosis was unavailable in children diagnosed before 2008. To study the effect of missing exposure data (due to lack of ID number for linkage, which was more common from 2004 to 2007), we assessed the association by year of dispense.

A 95% CI not overlapping the null value 1.00 for the OR was regarded as statistically significant. All analyses were performed by using Stata V.16.1 statistical software (StataCorp).

## Results

**Figure 1:**
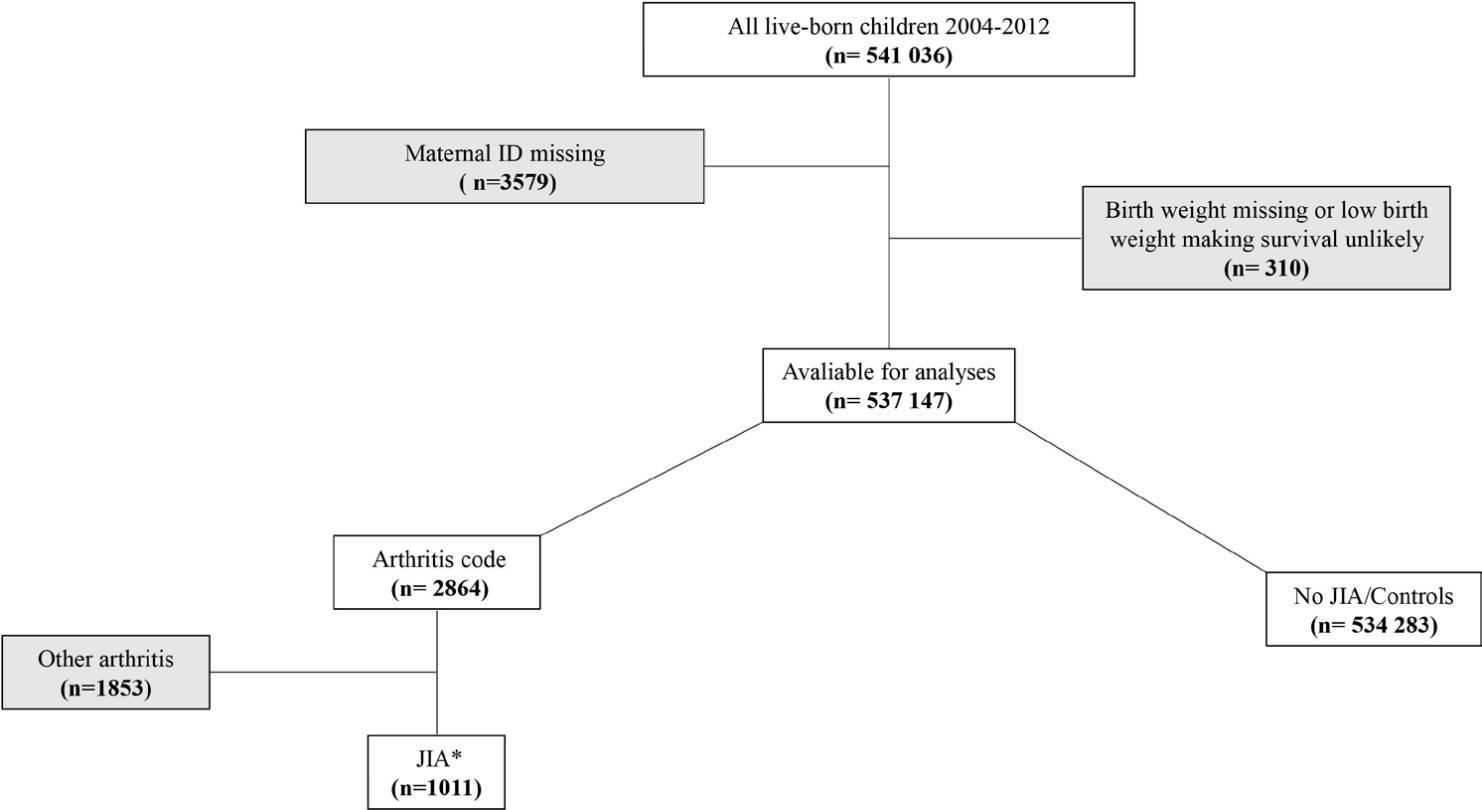
Flow chart of participants in main analyses. *≥2 M08, ≥M09 or 1 M08 and 1 M09. ≥1 M08 or ≥1 M09 if year of onset was 2020. ID, identification; JIA, juvenile idiopathic arthritis.

In the final analyses, 535 294 children and 360 769 mothers were included (figure 1). We identified 1011 JIA cases with 616 girls (60.1%) and 395 boys (39.1%) after a median follow-up (from birth through 31 december 2020) of 12.4 years (range 8.0–16.9). Of all children included, 149 534 (27.9%) were exposed to systemic antibiotics prenatally and 236 340 (44.2%) during 0–24 months of age (table 1). The distribution of selected baseline characteristics and covariates by dispensed antibiotics during 0–24 months of age are also presented in table 1.

**Table 1:** Distribution of selected baseline characteristics and potential confounders by dispensed systemic antibiotics during 0–24 months of life

	Dispensed systemic antibiotics during 0–24 months of age, n (%)	
Total	Yes, 236 340 (44.2)	No, 298 954 (55.9)
Sex		
Boys	128 672 (46.9)	145 937 (53.1)
Girls	107 668 (41.3)	153 017 (58.7)
Birth weight (grams)		
<2500	14 242 (54.7)	11 774 (45.3)
≥2500–4500	213 777 (43.5)	277 247 (56.5)
≥4500	8321 (45.6)	9933 (54.4)
Season of birth		
Winter (December–February)	55 182 (44.1)	69 933 (55.9)
Spring (March–May)	62 135 (45.1)	75 617 (54.9)
Summer (June–August)	63 127 (44.1)	79 874 (55.9)
Autumn (September–November)	55 896 (43.2)	73 530 (56.8)
Year of birth		
2004	24 929 (43.7)	32 084 (56.3)
2005	25 616 (45.1)	31 156 (54.9)
2006	22 602 (38.6)	35 893 (61.4)
2007	25 758 (44.2)	32 572 (55.8)
2008	27 221 (45.0)	33 300 (55.0)
2009	29 145 (47.0)	32 842 (53.0)
2010	29 123 (47.3)	32 392 (52.7)
2011	27 134 (45.0)	33 184 (55.0)
2012	24 812 (41.1)	35 531 (58.9)
Mode of delivery		
Vaginal birth	192 686 (43.3)	252 868 (56.8)
Elective caesarean section	16 112 (46.6)	18 475 (53.4)
Acute caesarean section	27 358 (50.0)	27 367 (50.0)
Unspecified caesarean section	184 (43.0)	244 (57.0)
Pre-eclampsia		
Yes	9193 (48.5)	9756 (51.5)
No	227 147 (44.0)	289 198 (56.0)
Maternal education level*		
Compulsory education	39 018 (44.6)	48 528 (55.4)
Medium education	70 549 (46.0)	82 780 (54.0)
Higher education	118 719 (43.5)	154 133 (56.5)
Missing	8054 (37.3)	13 513 (62.7)
Maternal age (years)		
<25	39 225 (44.4)	49 150 (55.6)
25–35	154 504 (44.7)	190 963 (55.3)
≥35	42 611 (42.0)	58 841 (58.0)
Maternal parity		
0	100 699 (44.6)	124 826 (55.4)
1	85 984 (45.0)	105 134 (55.0)
≥2	49 657 (41.9)	68 994 (58.2)
Maternal smoking in pregnancy		
Yes	16 545 (45.1)	20 172 (54.9)
No	179 056 (44.1)	226 679 (55.9)
Missing	40 739 (43.9)	52 103 (56.1)

Sum percentages are not 100 due to rounding.

There were significant differences (p<0.01) in antibiotic exposure status of all characteristics included.

*Compulsory education is 10 years. Medium length is an additional 4 years of vocational education or 3 years of general education that leads to qualification for admission to a university or other higher education. Higher education is education beyond medium length.

### Antibiotic exposure and diagnosed JIA

We found no significant association between prenatal exposure to antibiotics and JIA (aOR 1.10, 95% CI 0.96 to 1.26). In contrast, exposure to systemic antibiotics during 0–24 months of age was positively associated with JIA (aOR 1.40, 95% CI 1.24 to 1.59). The association was dose-dependent with a stronger association for increasing the number of dispensed courses. Subanalyses on different age periods showed significant associations in all age periods except for systemic antibiotics during 0–6 months, including the neonatal period (table 2).

**Table 2:** Association between antibiotic exposure and juvenile idiopathic arthritis (JIA)

	JIA	No JIA	OR (95% CI)	aOR* (95% CI)	P value†
	n=1011	n=534 283			
	n (%)	n (%)			
Prenatal exposure					
No exposure	709 (70.1)	385 051 (72.1)	Ref.	Ref.	
Any exposure	302 (29.9)	149 232 (27.9)	1.10 (0.96 to 1.26)	1.10 (0.96 to 1.26)	0.17
Third trimester	140 (13.9)	71 667 (13.4)	1.04 (0.87 to 1.24)	1.04 (0.87 to 1.24)	0.69
Repeated prenatal exposure					
No exposure	709 (70.1)	385 051 (72.1)	Ref.	Ref.	
1	205 (20.3)	99 494 (18.6)	1.12 (0.96 to 1.31)	1.12 (0.96 to 1.31)	0.16
≥ 2	97 (9.6)	49 738 (9.3)	1.06 (0.85 to 1.31)	1.06 (0.85 to 1.32)	0.60
Exposure during 0–24 months of age					
No exposure	487 (48.2)	298 467 (55.9)	Ref.	Ref.	
Any exposure	524 (51.8)	235 816 (44.1)	1.36 (1.20 to 1.54)	1.40 (1.24 to 1.59)	<0.001
Per course			1.07 (1.05 to 1.09)	1.08 (1.06 to 1.09)	<0.001
Repeated exposure during 0–24 months of age					
No exposure	487 (48.2)	298 467 (55.9)	Ref.	Ref.	
1	251 (24.8)	119 684 (22.4)	1.29 (1.10 to 1.50)	1.31 (1.13 to 1.53)	<0.001
≥ 2	273 (27.0)	116 132 (21.7)	1.44 (1.24 to 1.67)	1.50 (1.29 to 1.74)	<0.001
Any exposure in different age periods‡					
No exposure	487 (48.2)	298 467 (55.9)	Ref.	Ref.	
Neonatal period	36 (3.6)	17 267 (3.2)	1.28 (0.91 to 1.79)	1.34 (0.95 to 1.87)	0.09
0–6 months	65 (6.4)	35 123 (6.6)	1.13 (0.88 to 1.47)	1.19 (0.92 to 1.54)	0.20
0–12 months	195 (19.3)	98 246 (18.4)	1.22 (1.03 to 1.44)	1.26 (1.07 to 1.49)	0.006
6–12 months	147 (14.5)	71 934 (13.5)	1.25 (1.04 to 1.50)	1.30 (1.08 to 1.57)	0.006
12–24 months	452 (44.7)	188 514 (35.3)	1.47 (1.29 to 1.67)	1.51 (1.33 to 1.72)	<0.001
Age at first exposure§					
No exposure	487 (48.2)	298 467 (55.9)	Ref.	Ref.	
0–6 months	65 (6.4)	35 123 (6.6)	1.13 (0.88 to 1.47)	1.19 (0.92 to 1.54)	0.20
6–12 months	130 (12.9)	63 123 (11.8)	1.26 (1.04 to 1.53)	1.31 (1.07 to 1.59)	0.007
12–24 months	329 (32.5)	137 570 (25.8)	1.47 (1.27 to 1.69)	1.50 (1.30 to 1.72)	<0.001

*Adjusted for sex.

†P value for adjusted model.

‡Reference in each analyses is no dispensed antibiotic 0–24 months of age. One child may be exposed in several age periods.

§Reference in each analyses is no dispensed antibiotic 0–24 months of age.

aOR, adjusted OR.

The frequencies of different types of antibiotics are presented in table 3.

**Table 3:** Frequencies of subtypes of antibiotics dispensed during 0–24 months of age

Subtype of antibiotics*	ATC code	n	%†
Beta-lactamase sensitive penicillins	J01CE	193 262	40.5
Extended-spectrum penicillins	J01CA	119 836	25.1
Macrolides, lincosamides and streptogramins	J01F	117 853	24.7
Sulfonamides and trimethoprim	J01E	31 015	6.5
Systemic antibiotics not included in other subgroups‡	J01	15 523	3.3
Total§		477 489	100

*Parenteral antibiotics in the neonatal period not included (9181 individuals exposed only in the neonatal period).

†Sum percentages are not 100 due to rounding.

‡This group includes tetracyclines (J01AA), beta-lactamase-resistant penicillins (J01CF), combinations of penicillins including beta-lactamase inhibitors (J01CR), cephalosporins (J01DB-D), aminoglycosides antibacterials (J01G), quinolone antibacterials (J01M), glycopeptide antibacterials (J01XA), polymyxins (J01XB), steroid antibacterials (J01XC01), imidazole derivatives (J01XD), nitrofuratoin (J01XE), other antibacterials (J01XX).

§One child may receive several antibiotics during 0–24 months of age.

ATC, Anatomic Therapeutic Classification.

Subanalyses of different types of antibiotics showed a tendency towards a stronger association with sulfonamides/trimethoprim and the group containing more broad-spectrum antibiotics (‘systemic antibiotics not included in other subgroups’) (table 4).

**Table 4:** Association between exposure to subtypes of antibiotics during 0–24 months of age and juvenile idiopathic arthritis (JIA)

Subtype of antibiotics*	OR† (95% CI)	P value
Beta-lactamase sensitive penicillins	1.59 (1.36 to 1.86)	<0.001
Extended-spectrum penicillins	1.40 (1.14 to 1.72)	0.001
Macrolides, lincosamides and streptogramins	1.67 (1.38 to 2.02)	<0.001
Sulfonamides and trimethoprim	2.27 (1.55 to 3.32)	<0.001
Systemic antibiotics not included in other subgroups‡	2.50 (1.48 to 4.23)	0.001

*Parenteral antibiotics in the neonatal period not included.

†OR for JIA with 95% CI adjusted for sex, with cluster correction on individual children to correct for multiple exposures when comparing types of antibiotics dispensed to no antibiotic exposure during 0–24 months of age.

‡This group includes tetracyclines (J01AA), beta-lactamase-resistant penicillins (J01CF), combinations of penicillins including beta-lactamase inhibitors (J01CR), cephalosporins (J01DB-D), aminoglycosides antibacterials (J01G), quinolone antibacterials (J01M), glycopeptide antibacterials (J01XA), polymyxins (J01XB), steroid antibacterials (J01XC01), imidazole derivatives (J01XD), nitrofuratoin (J01XE), other antibacterials (J01XX).

The association between exposure to systemic antibiotics during 0–24 months of age and JIA was robust and essentially unchanged after adjustment for other potential confounders (table 5).

**Table 5:** Association between antibiotic exposure and juvenile idiopathic arthritis adjusted for child’s sex with additional adjustments for other potential confounders

	aOR (95% CI)	P value
Antibiotic exposure during 0–24 months adjusted for sex	1.40 (1.24 to 1.59)	<0.001
Additional adjustment for:		
Prenatal antibiotic exposure	1.40 (1.23 to 1.58)	<0.001
Season of birth*	1.40 (1.24 to 1.59)	<0.001
Year of birth	1.41 (1.25 to 1.60)	<0.001
Mode of delivery	1.40 (1.24 to 1.59)	<0.001
Birth weight, categorical†	1.40 (1.24 to 1.59)	<0.001
Pre-eclampsia	1.40 (1.24 to 1.58)	<0.001
Maternal age, categorical‡	1.40 (1.24 to 1.58)	<0.001
Maternal parity, categorical§	1.40 (1.24 to 1.58)	<0.001
Maternal education level¶	1.40 (1.24 to 1.59)	<0.001
Maternal smoking in pregnancy**	1.38 (1.21 to 1.59)	<0.001

*Winter=December–February, spring=March–May, summer=June–August, autumn=September–November.

†Birth weight in grams: <2500, ≥2500–4500, ≥4500.

‡Maternal age in years: <25, 25–35, ≥35.

§Maternal parity: 0, 1, ≥2.

¶Compulsory education is 10 years. Medium length is an additional 4 years of vocational education or 3 years of general education, that leads to qualification for admission to a university or other higher education. Higher education is education beyond medium length. Missing info in 21 567 children. Unadjusted OR with the same sample was 1.41 (1.24–1.59).

**Missing info in 92 842 pregnancies. Unadjusted OR with the same sample was 1.38 (1.21–1.59).

In sensitivity analyses excluding children with JIA born 2004–2006 and other children with JIA onset before 3 years of age (online supplemental figure 1), the association with childhood antibiotics also remained, though slightly attenuated (online supplemental table S1). Sensitivity analyses performed to study the effect of missing exposure data showed the strongest association during 2010–2013, the period with the most complete exposure data (online supplemental table S2).

10.1136/rmdopen-2023-003333.supp1 Supplementary data

10.1136/rmdopen-2023-003333.supp2 Supplementary data

### Discussion

In this nationwide register-based cohort study including more than 500 000 children and over 1000 JIA cases, we found that exposure to systemic antibiotics in early life was positively associated with diagnosed JIA. In contrast, we found no association between prenatal exposure to antibiotics and JIA development.

Our first main finding of an association between antibiotic exposure in early life and JIA is consistent with earlier studies,^15–18^ including a large register-based study from Finland in which JIA was defined by reimbursement codes for antirheumatic drugs.^16^ The authors did not describe whether non-steroidal anti-inflammatory drugs (NSAIDs) was included in the case-definition,^16^ which might have introduced a selection bias towards more severe cases since some children with JIA only receive first line treatments with NSAIDs and intra-articular corticosteroid injections.^22^ A strength of our study was a longer follow-up time, and we were able to adjust for relevant possible confounders.^16^

We found a dose-dependent association with a stronger association for an increasing number of dispensed courses, which is also in line with earlier findings.^15–18^ Subanalyses of different types of antibiotics showed a tendency towards a stronger association between sulfonamides/trimethoprim and the more broad-spectrum antibiotics and JIA. A similar observation in the Finnish study was that antibiotics targeting anaerobic organisms were stronger associated with JIA compared to antibiotics limited to aerobes. These findings were not replicated in the studies from the UK^15^ and Sweden,^17^ but small sample sizes^15 17^ and antibiotic exposure based on parental reporting^17^ might have limited the ability to show such differences.

Our second main finding was the observation of no association between prenatal exposure to antibiotics and JIA. This association has previously only been investigated in the All Babies in Southeast Sweden (ABIS) study, suggesting an association between antibiotic exposure in pregnancy with the development of systemic-onset JIA (n=7), but not with JIA as a whole group or with other subtypes of JIA.^17^

It is believed that changes in the gut microbiota may play an important role in the pathogenesis of JIA.^6 8 13 23^ If there is a causal link between antibiotics and JIA via perturbations on the gut microbiota, our results correspond well with studies showing that exposure to systemic antibiotics in early life can have a long-lasting influence on the composition and diversity of the gut microbiota of the child.^12^ The findings of a dose–response relationship and a tendency towards a stronger association with more broad-spectrum antibiotics may also support a causal connection between the antibiotic’s disruptive effect on the microbiota and JIA, as repeated antibiotic courses can have cumulative effects on the microbiome, and broad-spectrum antibiotics may have a more profound influence on the microbiota.^12^

Our findings do not prove any causality, and an alternative explanation to the observed association between antibiotic exposure in early life and JIA could be that children who later develop JIA have inherent higher risk of infections/are more vulnerable to infections. A reverse causality cannot be ruled out, as joint symptoms could be misinterpreted as bacterial infections.^24^ Our sensitivity analyses do, however, not support the notion of reverse causality. To minimise the risk of reverse causality, we defined the exposure window to the first 2 years of life where the risk of developing JIA is low. Since the most common subgroups of JIA have a biphasic peak of onset with the first peak already between 2 and 4 years,^7^ and we only knew the year of JIA onset, we also performed sensitivity analyses restricted to children with JIA with known age of onset after the age of 3 years. The associations remained, though slightly attenuated.

Strengths in our study were our design with a nationwide register-based study including all births in a defined time period, making it representative at a population level and reducing the risk of selection bias.^25^ Compared with most other studies, we had a virtually complete sample size. All data were collected prospectively with objective measures from high-quality population-based registries, leading to minimal risk of misclassification due to recall bias.^25^ We had a long follow-up, covering the whole age period <16 years for the oldest children in the study. The design minimises loss to follow up,^25^ which would only occur if a child with JIA was not registered in the NPR in case of moving out of Norway or not diagnosed correctly. This small risk of misclassification would however change the estimates minimally, due to the size of the cohort.

Our study is the first large scale, register-based study investigating prenatal antibiotic exposure with the risk of JIA. We were able to adjust for prenatal antibiotic exposure in our main analyses investigating the effect of antibiotic exposure in early life and risk of JIA, and the associations remained almost unchanged. These observations suggest that the association between antibiotics in early life and JIA is unlikely to be confounded by shared family factors. We were also able to adjust for many other possible confounders including socioeconomic status, which has often been lacking in previous studies.^5^ In addition, we had data on different types of antibiotics and different age periods including exposure in the neonatal period.

A limitation is the lack of data on infections as a potential confounder. However, previous studies have not been able to show any clear connection between infections and JIA,^6^ and our findings are also consistent with a case-control study from UK^15^ and a study from the ABIS cohort in Sweden,^17^ which had the advantage of adjusting for infections. Since JIA consists of different subtypes considered different diseases with different pathogenesis,^26^ information about subtypes would be desirable. In our study, approximately 30% of the prescriptions in NorPD from the first year of life lacked ID numbers (online supplemental table S2), precluding individual-level linkage and leading to misclassification of some children exposed to antibiotics as unexposed. Subanalyses on year of dispense show the strongest association between antibiotics and JIA during 2010–2013, the period with highest exposure accuracy, suggesting that missing exposures bias the overall results towards the null. Also the lack of association with exposure during 0–6 months could be explained by missing ID-numbers from NorPD, which was most common in this age period. Consequently, children in this age group were more often misclassified as unexposed to antibiotics. The statistical power was lower for early exposures, and the overlap of CIs should also call for cautious interpretation.

Except in the neonatal period, we had no data on systemic antibiotics administered in hospital. Nevertheless, if an infection requires hospitalisation, the treatment is often completed after discharge, and therefore registered in the NorPD. An exception can be antibiotic treatment given in the neonatal ward, but here we had data available from MBRN.

Another limitation is the lack of validation in the outcome definition, which could potentially lead to misclassification of the JIA cases. It is known that errors in medical coding occur,^27^ and we, therefore, required ≥2 ICD-10-codes in our case definition, since we consider it less likely that a majority of the children are coded incorrectly more than once.

In the search for causal risk factors in JIA, reference is often made to studies that investigated the gut microbiota in children with JIA demonstrating altered microbial composition.^23^ Since most of these studies are only cross-sectional, the causal link between these observations and JIA pathogenesis remains uncertain. Future research should focus on longitudinal studies including predisease microbiological samples, which would strengthen potential causality.

In this nation-wide register-based cohort study, we found an association with exposure to antibiotics in early life and JIA development which remained after adjustments, suggesting that this is an independent risk factor for JIA. The novel observation of no association with prenatal exposure to antibiotics suggests that the association between antibiotics in early life and JIA is unlikely to be confounded by shared family factors.

## Data Availability

Data are available on reasonable request. Data are not accessible due to national GDPR practices, but could be made available from the researchers on reasonable request.

## References

[1] Ravelli A, Martini A. Juvenile idiopathic arthritis. Lancet 2007;369:767–78. 10.1016/S0140-6736(07)60363-8 17336654

[2] Thierry S, Fautrel B, Lemelle I, et al. Prevalence and incidence of juvenile idiopathic arthritis: a systematic review. Joint Bone Spine 2014;81:112–7. 10.1016/j.jbspin.2013.09.003 24210707

[3] Berntson L, Andersson Gäre B, Fasth A, et al. Incidence of juvenile idiopathic arthritis in the Nordic countries. A population based study with special reference to the validity of the ILAR and EULAR criteria. J Rheumatol 2003;30:2275–82. 14528529

[4] Moe N, Rygg M. Epidemiology of juvenile chronic arthritis in northern Norway: a ten-year retrospective study. Clin Exp Rheumatol 1998;16:99–101. 9543575

[5] Horton DB, Shenoi S. Review of environmental factors and juvenile idiopathic arthritis. Open Access Rheumatol 2019;11:253–67. 10.2147/OARRR.S165916 31807094 PMC6842741

[6] Clarke SLN, Mageean KS, Maccora I, et al. Moving from nature to nurture: a systematic review and meta-analysis of environmental factors associated with juvenile idiopathic arthritis. Rheumatology (Oxford) 2022;61:514–30. 10.1093/rheumatology/keab627 34382060 PMC8824412

[7] Martini A, Lovell DJ, Albani S, et al. Juvenile idiopathic arthritis. Nat Rev Dis Primers 2022;8:5. 10.1038/s41572-021-00332-8 35087087

[8] Arvonen M, Vänni P, Sarangi AN, et al. Microbial orchestra in juvenile idiopathic arthritis: sounds of disarray Immunol Rev 2020;294:9–26. 10.1111/imr.12826 31833578

[9] Sarkar A, Yoo JY, Valeria Ozorio Dutra S, et al. The association between early-life gut Microbiota and long-term health and diseases. J Clin Med 2021;10:459. 10.3390/jcm10030459 33504109 PMC7865818

[10] Gensollen T, Iyer SS, Kasper DL, et al. How Colonization by Microbiota in early life shapes the immune system. Science 2016;352:539–44. 10.1126/science.aad9378 27126036 PMC5050524

[11] Kalbermatter C, Fernandez Trigo N, Christensen S, et al. Early life Colonization and breast milk drive immune development in the newborn. Front Immunol 2021;12:683022. 10.3389/fimmu.2021.683022 34054875 PMC8158941

[12] McDonnell L, Gilkes A, Ashworth M, et al. Association between antibiotics and gut Microbiome Dysbiosis in children: systematic review and meta-analysis. Gut Microbes 2021;13:1–18. 10.1080/19490976.2020.1870402 PMC7928022 33651651

[13] De Filippo C, Di Paola M, Giani T, et al. Gut Microbiota in children and altered profiles in juvenile idiopathic arthritis. J Autoimmun 2019;98:1–12. 10.1016/j.jaut.2019.01.001 30638708

[14] Kindgren E, Ahrens AP, Triplett EW, et al. Infant gut Microbiota and environment associate with juvenile idiopathic arthritis many years prior to disease onset, especially in genetically vulnerable children. EBioMedicine 2023;93:104654. 10.1016/j.ebiom.2023.104654 37329576 PMC10279551

[15] Horton DB, Scott FI, Haynes K, et al. Antibiotic exposure and juvenile idiopathic arthritis: A case-control study. Pediatrics 2015;136:e333–43. 10.1542/peds.2015-0036 26195533 PMC4516942

[16] Arvonen M, Virta LJ, Pokka T, et al. Repeated exposure to antibiotics in infancy: a predisposing factor for juvenile idiopathic arthritis or a sign of this group’s greater susceptibility to infections J Rheumatol 2015;42:521–6. 10.3899/jrheum.140348 25320218

[17] Kindgren E, Ludvigsson J. Infections and antibiotics during fetal life and childhood and their relationship to juvenile idiopathic arthritis: a prospective cohort study. Pediatr Rheumatol Online J 2021;19:145. 10.1186/s12969-021-00611-4 34530851 PMC8447683

[18] Räisänen LK, Kääriäinen SE, Sund R, et al. Antibiotic exposures and the development of pediatric autoimmune diseases: a register-based case-control study. Pediatr Res 2023;93:1096–104. 10.1038/s41390-022-02188-4 35854091 PMC10033398

[19] Irgens LM. Medical birth Registry--an essential resource in perinatal medical research. Tidsskr Nor Laegeforen 2002;122:2546–9. 12522882

[20] Bakken IJ, Ariansen AMS, Knudsen GP, et al. The Norwegian patient Registry and the Norwegian Registry for primary health care: research potential of two nationwide health-care registries. Scand J Public Health 2020;48:49–55. 10.1177/1403494819859737 31288711

[21] Størdal K, Mårild K, Blix HS. Use of antibiotics in children during the period 2005 – 16. Tidsskr Nor Laegeforen 2017;137:18. 10.4045/tidsskr.17.0272 28972334

[22] Grazziotin LR, Currie G, Twilt M, et al. Real-world data reveals the complexity of disease modifying anti-rheumatic drug treatment patterns in juvenile idiopathic arthritis: an observational study. Pediatr Rheumatol Online J 2022;20:25. 10.1186/s12969-022-00682-x 35410419 PMC8996666

[23] Xin L, He F, Li S, et al. Intestinal Microbiota and juvenile idiopathic arthritis: Current understanding and future prospective. World J Pediatr 2021;17:40–51. 10.1007/s12519-020-00371-3 32533534

[24] Thomas M, Bonacorsi S, Simon A-L, et al. Acute Monoarthritis in young children: comparing the characteristics of patients with juvenile idiopathic arthritis versus septic and undifferentiated arthritis. Sci Rep 2021;11:3422. 10.1038/s41598-021-82553-1 33564018 PMC7873238

[25] Thygesen LC, Ersbøll AK. When the entire population is the sample: strengths and limitations in register-based epidemiology. Eur J Epidemiol 2014;29:551–8. 10.1007/s10654-013-9873-0 24407880

[26] Zaripova LN, Midgley A, Christmas SE, et al. Juvenile idiopathic arthritis: from Aetiopathogenesis to therapeutic approaches. Pediatr Rheumatol Online J 2021;19:135. 10.1186/s12969-021-00629-8 34425842 PMC8383464

[27] Grytaas MA, Breivik L, Jørgensen AP, et al. The trouble with medical coding. Tidsskr Nor Laegeforen 2020;140. 10.4045/tidsskr.20.0541 33070589

