# Supplementary material for: Antibiotic exposure in prenatal and early life and risk of juvenile idiopathic arthritis: a nationwide register-based cohort study
Source: RMD Open. 2023 Aug 30;9(3):e003333. doi: 10.1136/rmdopen-2023-003333 (PMC10471866; doi:10.1136/rmdopen-2023-003333)
Supplement: Supplementary data [file rmdopen-2023-003333supp001.pdf]

Supplementary figure 1 Flowchart of participants in sensitivity analysis in cases with age of onset ≥3 years

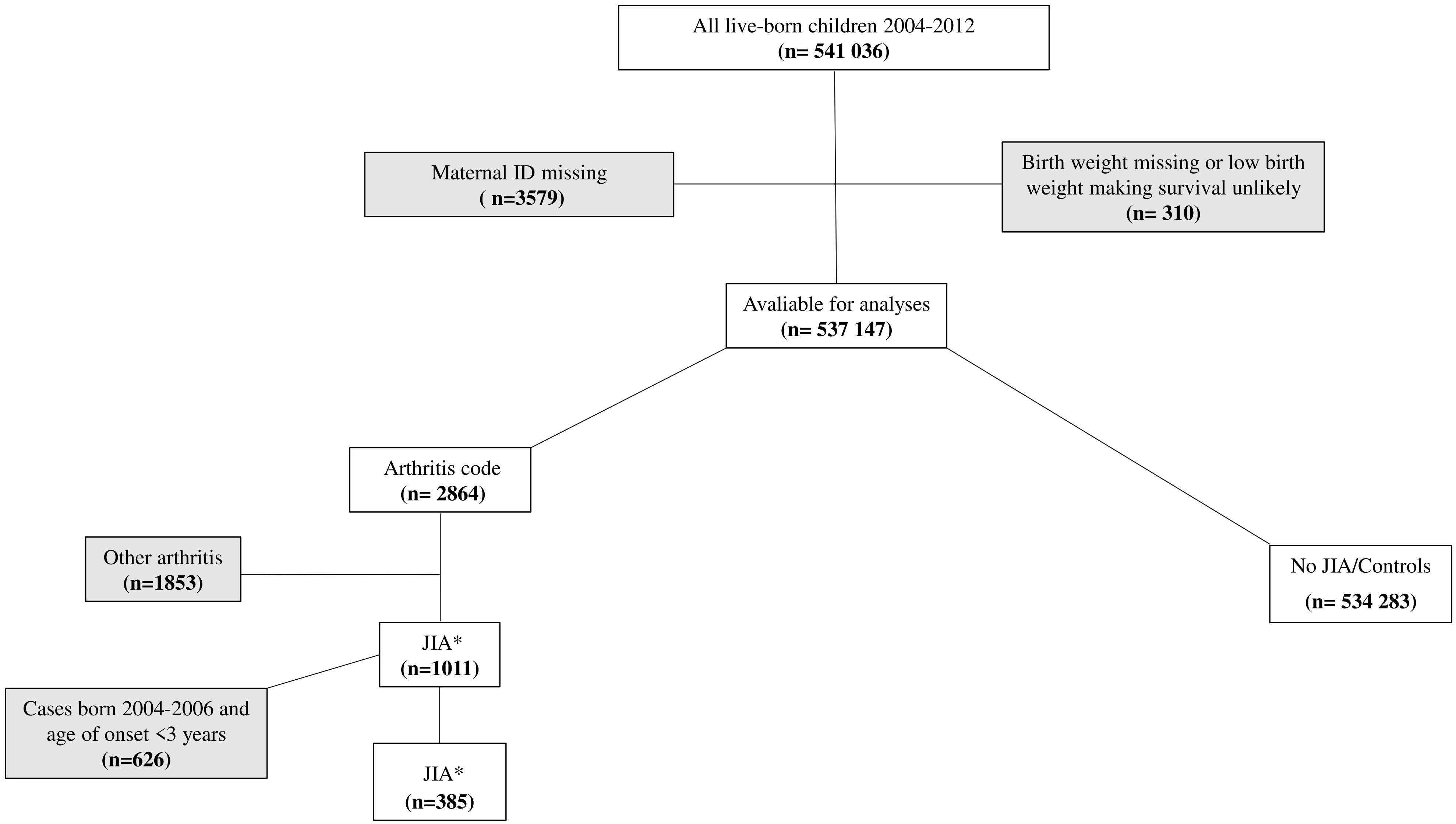

* ≥2 m08, ≥ m09, or 1m08 and 1m09. ≥ 1 m08 or m09 if year of onset was 2020
