# Supplementary material for: Antibiotic exposure in prenatal and early life and risk of juvenile idiopathic arthritis: a nationwide register-based cohort study
Source: RMD Open. 2023 Aug 30;9(3):e003333. doi: 10.1136/rmdopen-2023-003333 (PMC10471866; doi:10.1136/rmdopen-2023-003333)
Supplement: Supplementary data [file rmdopen-2023-003333supp002.pdf]

Supplementary table S1 Sensitivity analysis for the association between antibiotic exposure and juvenile idiopathic arthritis (JIA) in cases with age of onset ≥3 years

	JIA	No JIA	OR (95% CI)	aOR* (95% CI)
	Yes,<br>n=385	No,<br>n=534 283		
	n (%)	n (%)		
Prenatal exposure				
No exposure	262 (68.1)	385 051 (72.1)	Ref.	Ref.
Any exposure	123 (32.0)	149 232 (27.9)	1.21 (0.98-1.50)	1.21(0.98-1.50)
3. trimester	63 (16.4)	71 667 (13.4)	1.26 (0.97-1.65)	1.26(0.96-1.65)
Repeated prenatal exposure				
No exposure	262 (68.1)	385 051 (72.1)	Ref.	Ref.
1	83 (21.6)	99 494 (18.6)	1.23 (0.96-1.57)	1.23 (0.96-1.57)
2 or more	40 (10.4)	49 738 (9.3)	1.18 (0.85-1.65)	1.18 (0.85-1.65)
Exposure during 0-24 months of age				
No exposure	188 (48.8)	298 467 (55.9)	Ref.	Ref.
Any exposure	197 (51.2)	235 816 (44.1)	1.33 (1.09-1.62)	1.35 (1.11-1.66)
Per course			1.06 (1.03-1.09)	1.06 (1.04-1.09)
Repeated exposure during 0-24 months of age				
No exposure	188 (48.8)	298 467 (55.9)	Ref.	Ref.
1	104 (27.0)	119 684 (22.4)	1.38 (1.09-1.75)	1.40(1.10-1.78)
2 or more	93 (24.2)	116 132 (21.7)	1.27 (0.99-1.63)	1.30 (1.02-1.67)
Any exposure in different age periods†				
No exposure	188 (51.2)	298 467 (55.9)	Ref.	Ref.
0-6 months	30 (7.8)	35 123 (6.6)	1.36 (0.92-1.99)	1.39(0.94-2.05)
0-12 months	83 (21.6)	98 246 (18.4)	1.34 (1.04-1.74)	1.38(1.06-1.79)
6-12 months	58 (15.1)	71 934 (13.5)	1.28(0.95-1.72)	1.32(0.98-1.77)
12-24 months	154 (40.0)	188 514 (35.3)	1.30 (1.05-1.61)	1.32(1.06-1.63)
Age at first exposure‡				
No exposure	188 (51.2)	298 467 (55.9)	Ref.	Ref.
0-6 months	30 (7.8)	35 123 (6.6)	1.36 (0.92-1.99)	1.39(0.94-2.04)
6-12 months	53 (13.8)	63 123 (11.8)	1.33 (0.98-1.81)	1.37(1.00-1.86)
12-24 months	114 (29.6)	137 570 (25.8)	1.32 (1.04-1.66)	1.33(1.05-1.68)

Children with JIA born 2004-2006 and children born 2007-2012 with JIA onset < 3 years of age are excluded (n=626).
*Adjusted for sex
† Reference in each analyses is no dispensed antibiotic 0-24 months of age.
One child may be exposed in several age periods
‡ Reference in each analyses is no dispensed antibiotic 0-24 months of age

Supplementary table S2 Prescriptions lacking identification (ID) numbers the first year of life in the Norwegian Prescription Register

Year of dispense	Lacking ID number (%)	Antibiotics during 0-24 months of age stratified for birth year OR (95% CI)*
2004<br>2005<br>2006<br>2007	35<br>31<br>56<br>46	1.43 (1.21-1.68)
2008<br>2009	27<br>26	1.27 (0.97-1.68)
2010<br>2011<br>2012<br>2013	19<br>21<br>23<br>17	1.51 (1.16-1.98)

*OR with 95% confidence interval for Juvenile idiopathic arthritis when comparing children with ≥ 1 dispensed antibiotic during 0-24 months of age, to children with no dispensed antibiotics adjusted for sex with stratification on birth year
